# Carbolized Iodoform

**Published:** 1883-08

**Authors:** 


### Carbolized Iodoform.

The following formula is given by C. Sherk (*Berliner Klin. Wochenschrift*) as a great improvement over iodoform:

R.—Iodoform, 10 gr.

Acid, carbolic, 5 gr.

Ol. menth. pip., 2 drops.

The acid is to be rubbed up with the iodoform, and the peppermint oil added subsequently. The disagreeable odor of the drug is completely covered, and it is not again developed even at an elevated temperature.

